# Imaging Radial Distribution Functions of Complex Particles by Relayed Dynamic Nuclear Polarization

**DOI:** 10.1021/jacs.3c01279

**Published:** 2023-04-19

**Authors:** Pierrick Berruyer, Cynthia Cibaka-Ndaya, Arthur Pinon, Clément Sanchez, Glenna L. Drisko, Lyndon Emsley

**Affiliations:** † Institut des Sciences et Ingénierie Chimiques, Ecole Polytechnique Fédérale de Lausanne (EPFL), Lausanne CH-1015, Switzerland; ‡ Université de Bordeaux, CNRS, Bordeaux INP, ICMCB, UMR 5026, Pessac F-33600, France; § Swedish NMR Center, Department of Chemistry and Molecular Biology, University of Gothenburg, Gothenburg 41390, Sweden; ∥ Sorbonne Université, CNRS, Collège de France, UMR 7574, Chimie de la Matière Condensée de Paris, Paris F-75005, France; ⊥ Institute for Advanced Study (USIAS), University of Strasbourg, Strasbourg 67083, France; # University of Bordeaux, Pessac F-33600, France

## Abstract

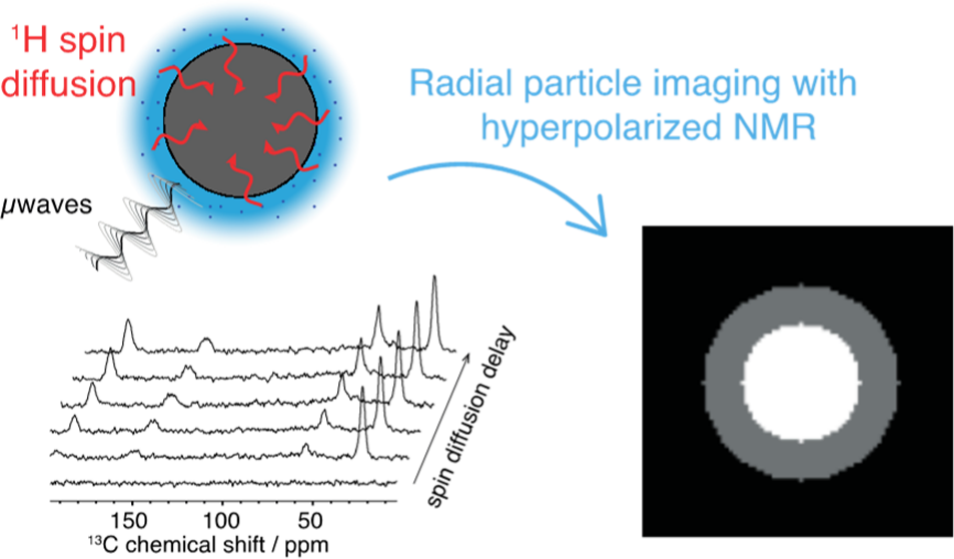

The physical properties of many modern multi-component materials are determined by their internal microstructure. Tools capable of characterizing complex nanoscale architectures in composite materials are, therefore, essential to design materials with targeted properties. Depending on the morphology and the composition, structures may be measured by laser diffraction, scattering methods, or by electron microscopy. However, it can be difficult to obtain contrast in materials where all the components are organic, which is typically the case for formulated pharmaceuticals, or multi-domain polymers. In nuclear magnetic resonance (NMR) spectroscopy, chemical shifts allow a clear distinction between organic components and can in principle provide the required chemical contrast. Here, we introduce a method to obtain radial images of the internal structure of multi-component particles from NMR measurements of the relay of nuclear hyperpolarization obtained from dynamic nuclear polarization. The method is demonstrated on two samples of hybrid core–shell particles composed of a core of polystyrene with a shell of mesostructured silica filled with the templating agent CTAB and is shown to yield accurate images of the core–shell structures with a nanometer resolution.

## Introduction

The physical properties of many modern nanocomposites and hybrid materials are determined by their internal microstructure.^1,2^ Performance can be significantly enhanced by optimizing domain size, chemical composition, and hierarchical porosity.^3,4^ Nanoarchitecture particles for theranostics,^5,6^ piezoelectric mesostructured particles,^7^ sponge-like catalysts for water splitting,^8^ smart fillers in polymer composites and in self-healing materials,^9,10^ and photonic crystals and glasses^11,12^ are few examples of applied materials dependent on the internal nanostructure. Thus, tools capable of characterizing these complex nanoscale architectures in composite multi-component materials are essential to design structures with targeted properties.

Depending on the morphology and the composition, domain sizes may be measured by laser diffraction, scattering methods, or by electron microscopy.^13−15^ However, these techniques sometimes fail to provide contrast between different components, for instance when all the components are organic, which is typically the case for formulated pharmaceuticals or multi-domain polymers. This lack of chemical contrast between molecular species is intrinsic to these methods that rely on, *e.g.*, electron density, which does not significantly vary from one organic molecule to another.

In nuclear magnetic resonance (NMR) spectroscopy, chemical shifts allow a clear distinction between components and can in principle provide the required chemical contrast. Moreover, although NMR is usually considered as a method to probe atomic-level local structures, in the solid-state the strong ^1^H dipolar coupling network offers an efficient way to probe much longer length scales. ^1^H spin diffusion experiments are the most widely used methods to assess domain sizes by NMR.^16−18^ In these experiments, an initial gradient of polarization is created, and the return to equilibrium driven by spin diffusion is monitored, providing information on domain sizes.^17,19−23^ More recently, the use of hyperpolarization from dynamic nuclear polarization (DNP) was demonstrated for domain size measurements.^24−27^ In this approach, denoted relayed DNP (R-DNP), hyperpolarization is used to generate a significantly higher polarization gradient across the sample, allowing access to nano- to micrometer domain size measurements.^28,29^ It has been used to measure the size of thin PEG layers adsorbed on organic crystalline drug nanoparticles,^30^ to characterize the structure of lipid nanoparticles (such as those used to deliver mRNA-based vaccines),^31^ to describe the topology of wood fibers,^32^ or to distinguish batch to batch variations in industrial hydroxypropylmethylcellulose ether samples.^33^ Although these NMR domain size measurements *via*^1^H spin diffusion and R-DNP have allowed a series of studies, some *a priori* knowledge of the internal structure and arrangement of the materials was required. NMR domain size measurements were typically used to refine an existing model of the material, usually assuming the position of the components and estimated sizes.

Here, we present an approach to obtain radial images of multi-component materials up to the micrometer length scale with a nanometer resolution, without the need to input any prior knowledge of the internal structure. Specifically, we introduce a method using measured hyperpolarization dynamics data to obtain the radial distribution functions of components of complex materials. The approach is demonstrated through the determination of the structures of two different sets of reference core–shell particles, composed of a polystyrene (PS) core, coated with a hybrid shell of silica and hexadecyltrimethylammonium bromide (CTAB).

## Results and Discussion

We first describe a formalism to extend the current description of R-DNP in order to obtain a radial image of materials having *K* components with an unknown distribution of the components within the material. Here, we assume that the system can be described with one variable in space because of symmetry. This would typically be the case for a flat surface (invariance to translation parallel to the plane defined by the surface), a cylinder (invariance to rotations around the cylinder axis and translation along the cylinder axis), and for spheres (invariance to rotations about any axis passing through the center of the frame). In the following section, we describe our formalism assuming a spherical symmetry (*i.e.*, spherical particles with a spherical distribution of the *K* components internally). Note that the formalism can be directly applied to a system with any of the abovementioned symmetries. Note also that radial images can be obtained from non-symmetrical objects but that interpretation is obviously more challenging and will not be discussed further here.

### Radial Imaging by Relayed DNP

**Figure 1:**
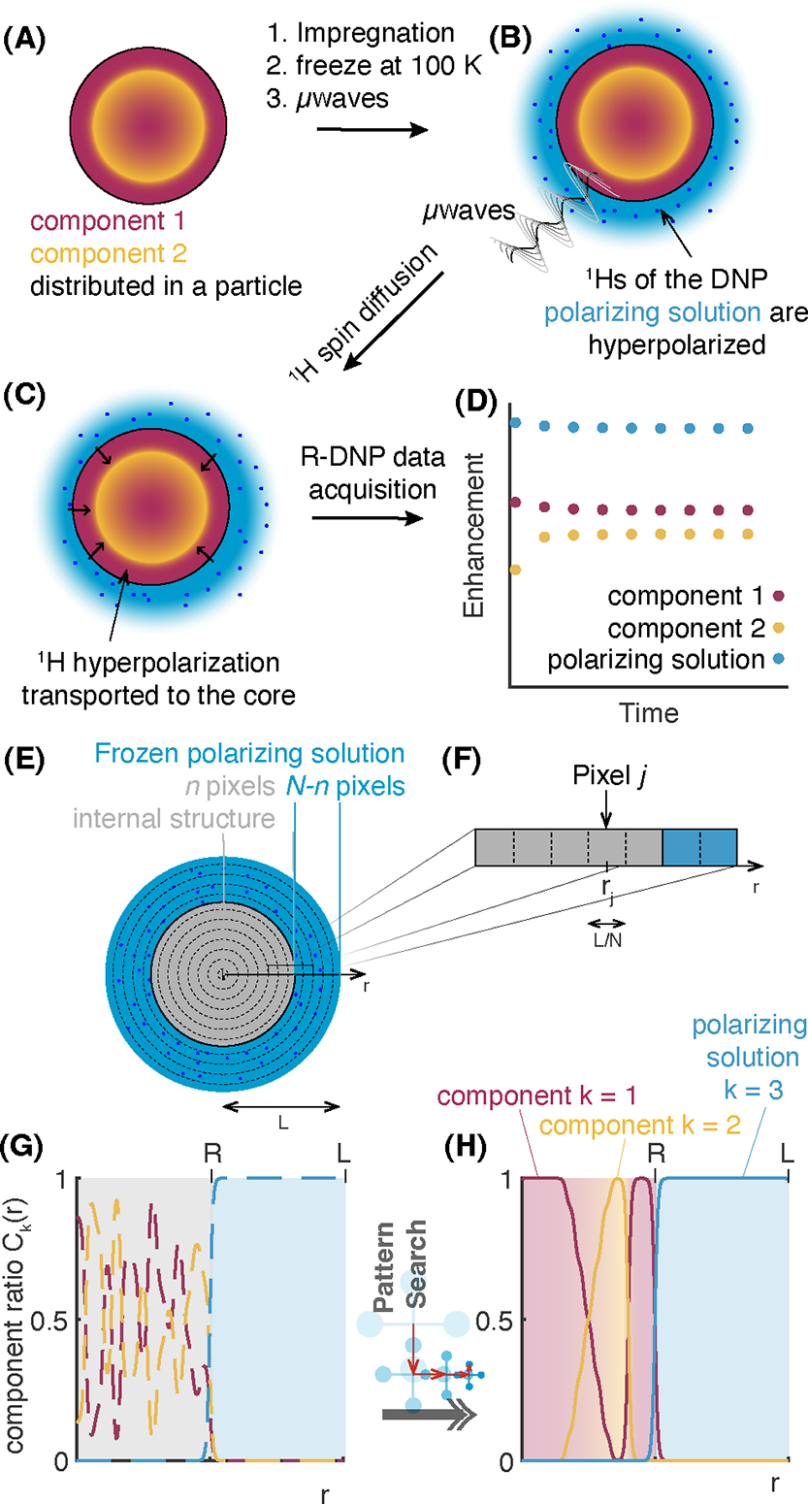
R-DNP radial imaging method. (a–d) Acquisition of the experimental data. A two-component spherical material (a) is impregnated with a non-solvent polarizing solution, which is then (b) cooled at 100 K to afford a frozen polarizing layer, the latter being hyperpolarized upon μwave irradiation. The hyperpolarization spontaneously transferred to the bulk material *via*^1^H spin diffusion (c). Finally, (d) the latter can be followed by measuring the DNP enhancement of the different components as a function of time. (e–h) Imaging procedure from experimental data. (e) Particle/solvent system is sliced into *N* concentric “voxels” (volume elements), in which the *n* spherical voxels closest to the center are the particle. (f) Details of the parametrization in voxels: *r*_*j*_ is the point at the center of the voxel *j*, of length *L*/*N*. (g) Local component ratio as a function of radius *r*. Initially, this local component ratio is discretized into pixels, with each pixel corresponding to a voxel inside the particle, and is given a random composition. (h) Local component ratio as a function of radius, *r*, after convergence.

Figure 1a–d summarizes the experimental procedure for radial imaging illustrated with an arbitrary composite particle with two chemical components (for simplicity of the representation) represented in Figure 1a in purple (component 1) and yellow (component 2) and which have some nonuniform radial distribution in the particle. To obtain experimental R-DNP data, the material is first impregnated with a polarizing solution (Figure 1b).^25,34^ Once impregnated the preparation is cooled to approx. 100 K, thus yielding a layer of frozen DNP polarizing solution at the surface of the particles. Upon μwave irradiation, the ^1^H nuclei in the polarizing solution are rapidly hyperpolarized by DNP. Then, the generated hyperpolarization spontaneously diffuses into the particle *via*^1^H spin diffusion, as shown in Figure 1c.

Using ^1^H saturation recovery experiments, the polarization build-up of the different components (components 1 and 2 and the DNP solvent) can then be monitored as a function of the polarization delay, with and without μwave irradiation of the sample, either directly in the ^1^H spectrum if the chemical shifts are resolved,^28^ or indirectly through transfer to ^13^C, ^15^N, or some other convenient probe nucleus.^35^ The DNP enhancement as a function of the polarizing time for the different components can then be determined by calculating the ratio between signal intensities with and without μwaves for each component (Figure 1d).

### Mathematical Description

The spontaneous transfer of hyperpolarization in the sample can be accurately described using thermodynamic models analogous to heat or mass transfer.^24,25,28,29^ Notably these models have been used to measure domain sizes in organic solids,^16,24,25,28^ including at high magnetic fields and fast magic-angle spinning (MAS), up to 60 kHz.^35^

The transport of DNP enhanced polarization by ^1^H spin diffusion in a multi-component particle can be described by solving the spin diffusion eq 1.^24,25,28,29^
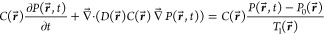
1where ***r⃗*** is the vector position, *t* is the time, *P*(***r⃗***, *t*) is the polarization at point ***r⃗*** and time *t*, *P*_0_(***r⃗***) is the local equilibrium polarization at point ***r⃗***, *D*(***r⃗***) is the ^1^H spin diffusion coefficient at position ***r⃗***, *T*_1_(***r⃗***) is the longitudinal relaxation time at position ***r⃗***, and *C*(***r⃗***) is the proton concentration at position ***r⃗***. Note that for simplicity the spin diffusion coefficient *D*(***r⃗***) is written as a position-dependent scalar and not as a position-dependent tensor because the spatial averaging of the microscopic spin diffusion tensor most of the time leads to a locally (almost) isotropic spin diffusion tensor on the nanometer scale relevant here.^36^

As laid out above, here we consider the case of spherical symmetry of the system (*i.e.* spherical particles with a spherical distribution of the *K* components). Thus, the vector position ***r⃗*** reduces to the distance from the center of particle *r*. Note that the symmetry argument reduces significantly the number of variables in the imaging procedure, and the approach can automatically be used for other symmetries such as a flat surface or a cylinder (by simply using the appropriate expressions for the vectorial operators in eq 1).

We now consider that the sample is made-up of *K* different components, where the relative content of component *k* (*k* ∈ ⟦1, *K*⟧) at point *r* is given by *C*_*k*_(*r*), then the characteristic build-up time as a function of position can be written as:  where *T*_1_^*k*^ is the build-up time of the component *k* isolated from dipolar contact with dissimilar spin reservoirs. This is the spin–lattice relaxation time of the dry individual components (at the same temperature and the same MAS rate as used for the R-DNP experiments) or, for the polarizing solution component, it is *T*_b_^DNP^, which is the build-up time under DNP of the pure polarizing solution.

We can also define the ^1^H spin diffusion coefficient as a function of *r*, , where *D*_*k*_ is the ^1^H spin diffusion coefficient of the pure phase of the given component *k*. The ^1^H concentration at point *r* is defined similarly, , where [^1^*H*]_*k*_ are the set of ^1^H concentrations in components *k*. Considering this formalism, eq 1 becomes
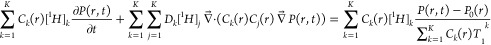
2

We now see that the polarization dynamics of each component will depend on *C*_*k*_(*r*). We can therefore determine the set of *C*_*k*_(*r*) from the ensemble of R-DNP curves as follows.

If we suppose that the *C*_*k*_(*r*) can be discretized into *N* pixels, we obtain a *K* x *N* matrix to describe the complete composition of the sample, where the *K* lines represent the different components, and the *N* columns gives the relative content in each pixel (from pixel 1 at *r* = 0 to pixel *N* at *r* = R). The first *n* pixels are from the solid particle and the following *N-n* are from the polarizing solution. By convention, we define the last component (*K*) to be the polarizing solution.
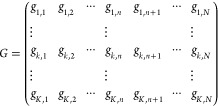
3

For simplicity, we will assume a clear separation between the solid particle and the polarizing solution so that the composition matrix can be simplified to
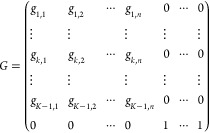
4

This assumption is made here to reduce the necessary computing time, but the formalism developed here is valid generally and thus allows, for instance, the solid–liquid interface to be modeled.

This description based on discrete radial pixelization can then be used to construct trial functions describing the ratio of each component as a function of *r* in the system. The R-DNP build-up curves (Figure 1d) can then be predicted for any given trial composition.

To do this, the pixel-by-pixel representation must be converted into a continuous function in order to solve eq 2. For instance, function *f*_*k*_ giving the ratio of component *k* at point *r* is
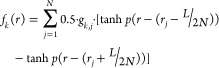
5where *p* is a constant defining the slope of a hyperbolic tangent function connecting each pixel, *r*_*j*_ is the point at the center of the pixel *j* whose composition in component k is given by *g*_*k,j*_, and *L* is the total length of the system (particle + DNP solution layer), as illustrated in Figure 1f.

Finally, the local equilibrium polarization function is defined as
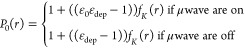
6where ε_0_ is the solvent DNP enhancement, ε_dep_ is the depolarization factor, and *f*_*K*_(*r*) is the function locating the polarizing solution (the component *K* is defined above by convention to be the polarizing solution).^37,38^ In a general way, we can write the DNP enhancement of the component k
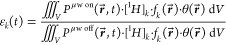
7

For a spherical symmetry (with spherical coordinates *r*, Θ, φ), it becomes
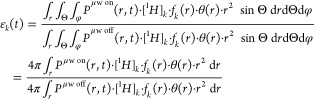
8

Thus, any particle composition described by a matrix (*G*) can be input to simulate the sample polarization and DNP enhancements as a function of time for each component of the system. θ(*r*) takes into account the polarization quenching or bleaching in the direct surroundings of the DNP polarizing agents (*i.e.*, in the frozen polarizing solution).

In particular, we can now iteratively optimize the compositions *C*_*k*_(*r*) in order to best match the experimental data and obtain radial images. To do this, we start from random starting compositions, as illustrated in Figure 1g, to simulate the DNP enhancements of all the components and the polarizing solution. We then minimize the RMSD between the simulated data and the experimental enhancements as a function of time, here using a pattern search algorithm. In principle, any minimization routine can be used, but the pattern search method was chosen as it can (i) handle a large number of variables, (ii) be parallelized and thus significantly speed up the process (here 28 computing cores were typically used), and (iii) efficiently explore a large number of parameter combinations. We also tested simplex method but, in our hands, it remains in very local minima, whereas the pattern search efficiently found more global minima (Figure 1h).

### Application of the R-DNP Radial Imaging Method to Hybrid Core–Shell Particles

**Figure 2:**
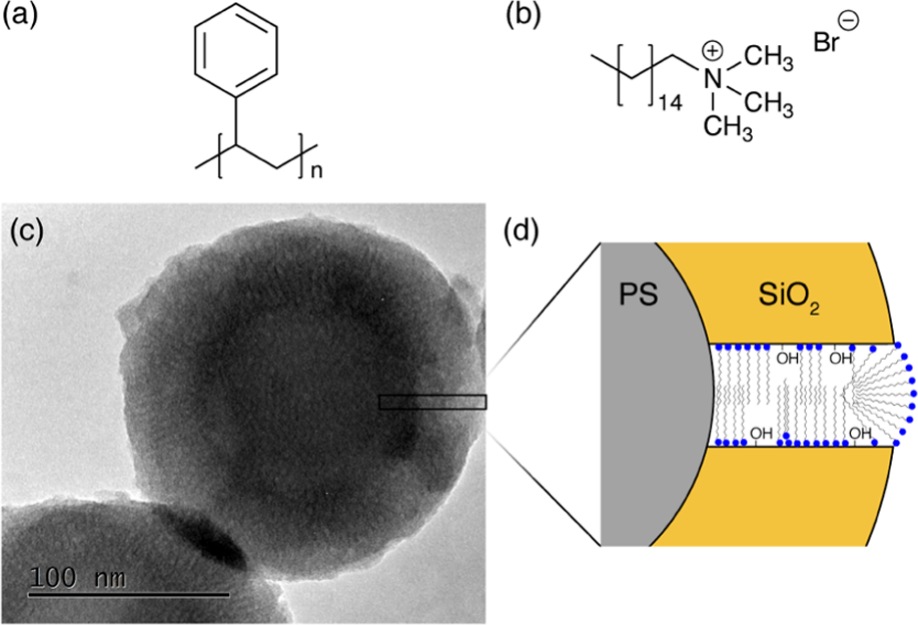
Molecular structure of (a) PS and (b) CTAB. (c) TEM image of sample A, composed of a PS core and a CTAB-templated silica shell, which presents radial mesoporosity. (d) Schematic of one radial pore templated by CTAB and the silanol terminations of the silica phase.

To demonstrate the applicability of the radial imaging method, an ideal model system was needed. This was found in core–shell particles, composed of a core of polystyrene and a shell of silica containing CTAB (Figure 2). The polystyrene latexes are monodispersed in size, providing a suitable core. Sol–gel chemistry is then used to deposit a shell of well-controlled thickness. The shell is mesostructured, and CTAB arranges itself into radial micelles starting from the polystyrene surface and continuously extending to the particle surface. As transmission electron microscopy (TEM) of the PS/SiO_2_ particles does not provide an excellent contrast (Figure 2c), the TEM image of calcined particles was recorded. Calcination removes the PS core and the CTAB to produced hollow silica particles.^39^ The TEM image of the hollow silica particles is reported in Figure S3 and provides a clearer visualization of the shell. Note that the low contrast of TEM on the original PS/SiO_2_ particles is typically one of the motivations to develop the NMR chemical shift-based R-DNP radial imaging method here. Beyond the well-defined core–shell design of these particles, PS and CTAB signals can be clearly resolved and provide the chemical contrast required to obtain an image (Figure S4), while CTAB and silanols provided a dense ^1^H network through the silica shell. As shown in ref (26), the DNP polarizing agent does not enter CTAB nanochannels. We synthesized two model samples: the size of the polystyrene core is kept constant and the silica/CTAB shell is varied to yield samples A and B with known structures (obtained from TEM measurements, see Figures S1 and S2), and the core and shell dimensions are given in Table 1.

**Table 1:** Experimental Domain Size of the PS Core and SiO_2_/CTAB Shell of Sample A and B, Measured with TEM and Radial Imaging R-DNP^a^

		measurement method	
sample		TEM	R-DNP
A	*r*_PS_ (nm)	46 ± 4	58 ± 2
	*d*_SiO2/CTAB_ (nm)	34 ± 9	22 ± 2
B	*r*_PS_ (nm)	46 ± 4	58 ± 6
	*d*_SiO2/CTAB_ (nm)	22 ± 8	10 ± 6

a The reported TEM error corresponds to the width of the size distribution obtained from picture analysis (see Figures S1 and S2). The R-DNP error is the error of the fit obtained from a Monte-Carlo analysis.

The samples for R-DNP measurements are prepared as described in the Materials and Methods section below. Both samples were impregnated with a solution containing 10 mM AMUPOL and 80 mM ^13^C-formate in glycerol-*d*_8_/H_2_O 6/4_v/v_. As described by Prisco *et al.*, ^12^C-glycerol-*d*_8_/H_2_O 6/4_v/v_ used here allowed to maximize the cooling power of the DNP radical solution.^29^Figure S4 shows the 9.4 T ^1^H–^13^C DNP CPMAS spectrum recorded on impregnated sample A, as compared to pure dry CTAB and PS under the same experimental conditions. The ^13^C-formate, PS, and CTAB signals can be clearly resolved to provide the chemical contrast required to obtain an image. Varying the polarization delay, the build-up of the signal as a function of time was measured under μwave irradiation: , , and , and without μwave irradiation: , , and . Experimental DNP enhancements for the different phases are then calculated with
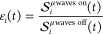
9

**Figure 3:**
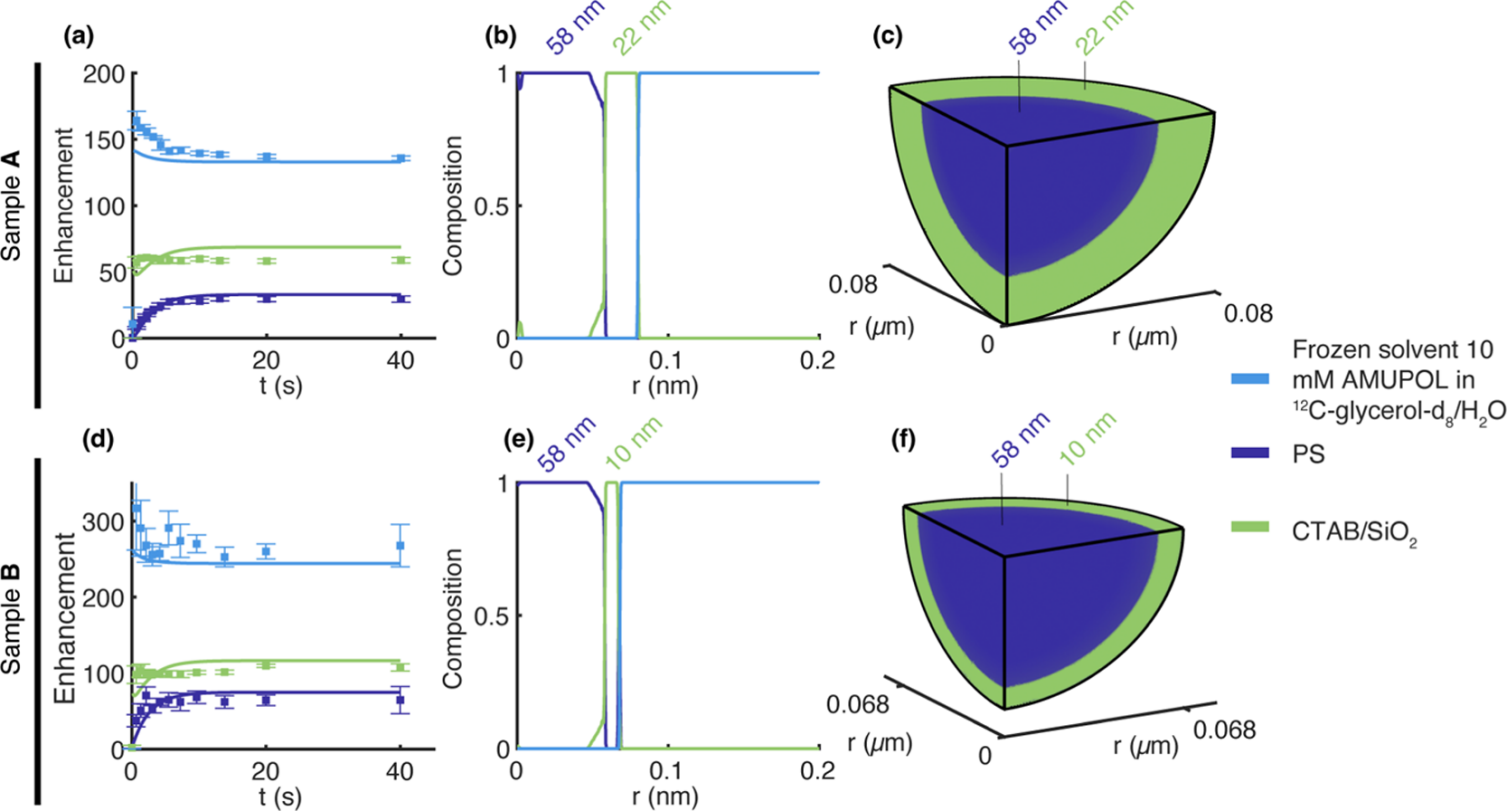
R-DNP radial imaging procedure applied to hybrid particles A and B (described in the text). (a,d) Experimental ^1^H DNP enhancements as a function of DNP polarization time (filled circles) measured for the three different phases of sample A (a) and sample B (d), and simulations (solid lines) from the best-fit radial images. (b,e) Best fit determined radial images of each component of sample A (b) and sample B (e). (c,f) 3D representation of the determined particle structure of sample A (c) and sample B (f). In all panels, dark blue refers to the PS, green is the CTAB/SiO_2_, and light blue refers to the frozen 10 mM AMUPOL solution in the ^12^C-glycerol-*d*_8_/H_2_O solvent.

The experimental data are shown in Figure 3a,d for samples A and B, respectively. In order to obtain images as described above, ^1^H spin–lattice relaxation times of the components are needed. The ^1^H spin–lattice relaxation time of SiO_2_/CTAB was measured on dry particles of porous silica spheres filled with the templating agent to mimic the SiO_2_/CTAB shell of the composite particles. At 9.4 T and 100 K with a MAS rate of 8 kHz, we found *T*_1_^SiO2/CTAB^ = 52 s. Under the same conditions, the ^1^H spin–lattice relaxation time of PS and the ^1^H build-up time of the pure DNP solvent phase *T*_b_^DNP^ are known from the literature to be *T*_1_^PS^ = 1.3 s and *T*_b_^DNP^ = 2.5 s.^29^ Finally, the CTAB and ^12^C-glycerol-*d*_8_/H_2_O 6/4_v/v_^1^H spin diffusion coefficients were calculated by scaling the known ^1^H spin diffusion coefficient of a static PS sample to account for the difference in ^1^H concentrations.^29^ The MAS dependence under the present experimental conditions has been described by Chaudhari *et al.* and is used to adapt for a MAS rate of 8 kHz.^40^ All the parameters used, together with the raw NMR data and MATLAB scripts, are given in the Supporting Information.

For sample A, the radius of the whole particle, as measured by TEM, is *R*_A_ = 80 nm. We therefore modelled the entire system (the particle with a separate impregnating AMUPOL/glycerol-*d*_8_/H_2_O layer, which we assume does not penetrate into the particle)^26^ by *N* = 200 voxels of 1 nm each, assuming spherical symmetry. The first *n* = 80 voxels (of the *N* = 200 pixels) model the hybrid particle, while the remaining 120 voxels represent the impregnating solution (Figure 1e). As laid out above, the composition of the *n* particle voxels are then varied iteratively to fit the DNP data and the composition converged to the structure shown in Figure 3b,c. While the initial starting guess is generated randomly and thus does not assume any particular shape for the internal structure, the fitting procedure converges to an image with a very clear core–shell structure with a PS core of 58 nm radius, covered by a 22 nm hybrid silica shell containing the template agent CTAB.

Similarly, for sample B, TEM indicates a total particle size of *R*_B_ = 68 nm, and we then used *N* = 200 voxels of 1 nm for the simulation, in which the first *n* = 68 voxels describe the hybrid particle. The results are shown in Figure 3d–f. Starting from a random internal composition, the final image (Figure 3e,f) again yields a very well-defined core–shell structure with a PS core of 58 nm radius, covered by a 10 nm CTAB/silica shell. The value of the core radius is reproduced well between the two samples (Table 1). Thus, the R-DNP radial imaging method, applied to samples A and B, yields internal particle structures that are in fair agreement with the TEM measurements.

Here, for both samples, A and B, the images were obtained by minimizing the RMSD only between the simulated and experimental DNP enhancements of PS, SiO_2_/CTAB, and the frozen polarizing solution. Figures S7 and S8 show the build-up of the signals as a function of time with and without microwaves (experimental and simulated). It should be possible to also include these signal build-ups as a function of time directly into the RMSD calculations, but we note that they are the components used to calculate the DNP enhancement, and optimizing the exact target function used will be the subject of future work.

### Error of the Convergence Procedure within Noise

To assess the convergence of the images, the error of the fit is evaluated using a Monte Carlo approach (see methods below). As shown in Figures S5 and S6, for both samples, we found small errors due to the fit, and these errors are mostly localized at the very center of the particles and the transition between the SiO_2_/CTAB regions. At the very center of the particles (*r* between 0 and 4 nm), there appears to be a standard deviation of the probability of up to 0.4 for the composition. Also, in the transition region between the PS and SiO_2_/CTAB phases, we observed a standard deviation in the composition of up to ±0.05 for sample A and ±0.5 for sample B. The profiles of the standard deviations for both samples, A and B, are provided in Figures S5 and S6. All in all, the convergence is found to be very robust. This analysis gives us the error of the fitting procedure in terms of composition for each pixel. Now, considering the border between the two regions, PS and SiO_2_/CTAB, we can translate the composition error per pixel into a domain size error. Those estimations have been reported in Table 1 and, again, confirm the robustness of the fit procedure.

## Conclusions

To conclude, we have introduced a method to obtain radial images of the internal structure of multi-component organic particles, using the dynamics of hyperpolarization as measured with R-DNP data and exploiting the chemical shift contrast provided by NMR. The method was demonstrated here on two samples of hybrid core–shell particles composed of a core of polystyrene with a shell of mesostructured silica filled with the templating agent CTAB. In both cases, we found PS cores and SiO_2_/CTAB dimensions in agreement with dimensions obtained independently by TEM. The technique can be extended to other nanoparticle systems, provided that a suitable impregnating formulation can be found. It thus provides a reliable and efficient method to image the internal structure of materials at the nanometer resolution on up to micron length scales. We expect this will be especially useful in cases where existing microscopy methods do not provide sufficient contrast. Typically, this might be the case, for example, for pharmaceutical amorphous solid dispersions, which are becoming increasingly important.^41^ Finally, the imaging method introduced here should become faster and more robust with the development of DNP at high magnetic fields and fast MAS (up to 65 kHz MAS), allowing ^1^H detected R-DNP imaging to be implemented.^35^

## Materials and Methods

### Materials Synthesis

Anionic latexes were prepared following the procedure from Blas *et al.*(39) Raw materials were obtained from Sigma-Aldrich and used without further purification. Dihexylsulfosuccinate sodium salt aqueous solution (2.65 g) was mixed with purified water (200 mL) and sodium bicarbonate (0.43 g). Styrene was passed through basic alumina. Styrene (30 g) was added to the solution at room temperature and then degassed by bubbling with argon for 30 min. Potassium persulfate (0.43 g) was dissolved in purified water (14.8 g) and then added *via* a syringe to the styrene solution. The reaction was placed in a pre-heated bath at 90 °C and stirred. After 5.5 h, the reaction was removed from the bath and then dialyzed over 6 days to remove excess surfactant. The colloidal solution was 9 wt % polystyrene.

The coating procedure applied followed the synthesis reported by Blas *et al.*.^39^ CTAB (3.2 g) was dissolved in 100 mL of purified water by gently heating the solution while agitating. Purified water (500 mL), absolute ethanol (200 mL), and 28% ammonium hydroxide solution (7.5 g) were mixed, generating a solution at pH 10.5. The polystyrene latexes (3 g) and the CTAB solution were added to the basic solution. The mixture was stirred for 30 min, then tetraethyl orthosilicate (5.4 g) was added dropwise while stirring vigorously. After 24 h, the particles were separated from the solution by centrifugation at 8000 rpm for 1 h and washed in water three more times using the same conditions. Finally, the prepared spheres were kept in a small amount of water (∼25 mL). The procedure used to obtain SiO_2_ particles with radial CTAB-templated pores followed the route described above for coating of PS spheres with silica except that the polystyrene latexes were not introduced to the preparation. The amount of TEOS used in this case was 10.8 g.

### NMR Sample Preparation

For R-DNP experiments, the core–shell hybrid particle suspension was centrifuged at 2000*g* during 30 s. The supernatant was then removed and the particles re-suspended in a solution (10 μL) of 10 mM AMUPol and 80 mM ^13^C-labeled sodium formate in H_2_O/^12^C-glycerol-*d*_8_: 4/6 v/v. A few lacquered KBr crystals were added to the suspension to allow sample temperature measurements. The sample was then transferred to a 3.2 mm sapphire rotor, sealed with a silicon plug, and capped with a zirconia drive cap. The filled DNP rotor was inserted into the pre-cooled (*ca.* 100 K) 3.2 mm LTMAS DNP NMR probe, where the sample was rapidly frozen within seconds.

### NMR Experiments

DNP experiments were performed on a 400 MHz Avance III Bruker DNP solid-state NMR spectrometer. The spectrometer was equipped with a LTMAS DNP 3.2 mm ^1^H/^13^C double resonance probe and a 263 GHz gyrotron capable of outputting *ca.* 5–10 W of CW microwaves. The main magnetic field was adjusted to match the maximum positive ^1^H DNP enhancement of the biradical agent AMUPol. DNP enhancements were determined by comparing the peak areas of the spectra acquired with and without μwave irradiation. Variable amplitude cross-polarization was used to transfer polarization from ^1^H to ^13^C. SPINAL-64 heteronuclear ^1^H decoupling with an rf field of 100 kHz was applied in all cases. The temperature was measured based on the *T*_1_(^79^Br) peak and carefully adjusted so that experiments with and without μwaves had the same internal sample temperatures.^42^ This was done to avoid any temperature-related DNP enhancement misinterpretation.^33,43^ All the NMR raw data is available in the Supporting Information.

### Matlab Calculations

Numerical simulations were performed using either the HPC facilities of the Scientific IT and Application Support Center of EPFL running MATLAB R2019b with 28 computing cores (Intel Xeon 2.6 GHz) on a single calculation node with 128 GB of RAM, or an Apple Mac Pro (2019) running MATLAB R2020b with 28 cores (Intel Xeon 2.5 GHz) and 192 GB of RAM. All the scripts used are available in the Supporting Information.

### Error Calculation

To assess the convergence of the images, the error of the fit was evaluated using a Monte Carlo approach. With the best fit, the fitting parameters were then used to generate a set of synthetic time-dependent enhancement data. 10% of random noise was then added to the generated data and used to perform a new fit. This procedure was repeated 10 times. Note that every time the procedure was repeated, the initial guess was different (randomly generated) and noise added to the data was different (random noise). Finally, the standard deviation of the 10 converged images was calculated to evaluate the error of the fit.

### TEM Experiments

The samples were prepared by depositing one drop of the colloidal dispersion onto a conventional carbon-coated copper grid. Grids were air dried at room temperature and stored in a closed box to prevent dust accumulating. TEM experiments were performed using a JEOL JEM 1400+ operating at 120 kV with a LaB6 filament.

## Supporting Information Available

Detailed experimental NMR parameters, TEM, size distribution, signal build-up, NMR raw data, Mat lab scripts (PDF)

## Notes

Raw data and Matlab scripts are available from Zenodo at the DOI: 10.5281/zenodo.7756308 under the license CC BY-SA 4.0 (Creative Commons Attribution-ShareAlike 4.0 International).
