# Supplementary material for: Imaging Radial Distribution Functions of Complex Particles by Relayed Dynamic Nuclear Polarization
Source: J Am Chem Soc. 2023 Apr 19;145(17):9700–7. doi: 10.1021/jacs.3c01279 (PMC10760979; doi:10.1021/jacs.3c01279)
Supplement: Supplementary file 1 — ja3c01279_si_001.pdf [file ja3c01279_si_001.pdf]

Supporting information

Imaging radial distribution functions of complex particles by relayed dynamic nuclear polarization

Pierrick Berruyer,[a,*] Cynthia Cibaka-Ndaya,[b] Arthur Pinon, Clément Sanchez,[d-f] Glenna L. Drisko,[b] and Lyndon Emsley[a *]

[a] Institut des Sciences et Ingénierie Chimiques, Ecole Polytechnique Fédérale de Lausanne (EPFL), CH-1015 Lausanne, Switzerland

[b] Université de Bordeaux, CNRS, Bordeaux INP, ICMCB, UMR 5026, F-33600 Pessac, France.France

[c] Swedish NMR Center, Department of Chemistry and Molecular Biology, University of Gothenburg, 41390 Gothenburg, Sweden

[d] Sorbonne Université, CNRS, Collège de France, UMR 7574, Chimie de la Matière Condensée de Paris, F-75005 Paris, France

[e] University of Strasbourg, Institute for Advanced Study (USIAS), 67083 Strasbourg,France

[f] University of Bordeaux, F-33600 Pessac, France

*corresponding authors: pierrick.berruyer@epfl.ch (PB); lyndon.emsley@epfl.ch (LE)

NMR RAW DATA AND MATLAB SCRIPT

The NMR raw data, and the Matlab scripts are available from Zenodo at the DOI: 10.5281/zenodo.7756308.

The README file includes the Zenodo deposit provide the full description of the deposited data.

Data and scripts are made available under the license CC BY-SA 4.0 (Creative Commons Attribution-ShareAlike 4.0 International). See licensing details at:
https://creativecommons.org/licenses/by-sa/4.0/

1. Supplementary figures

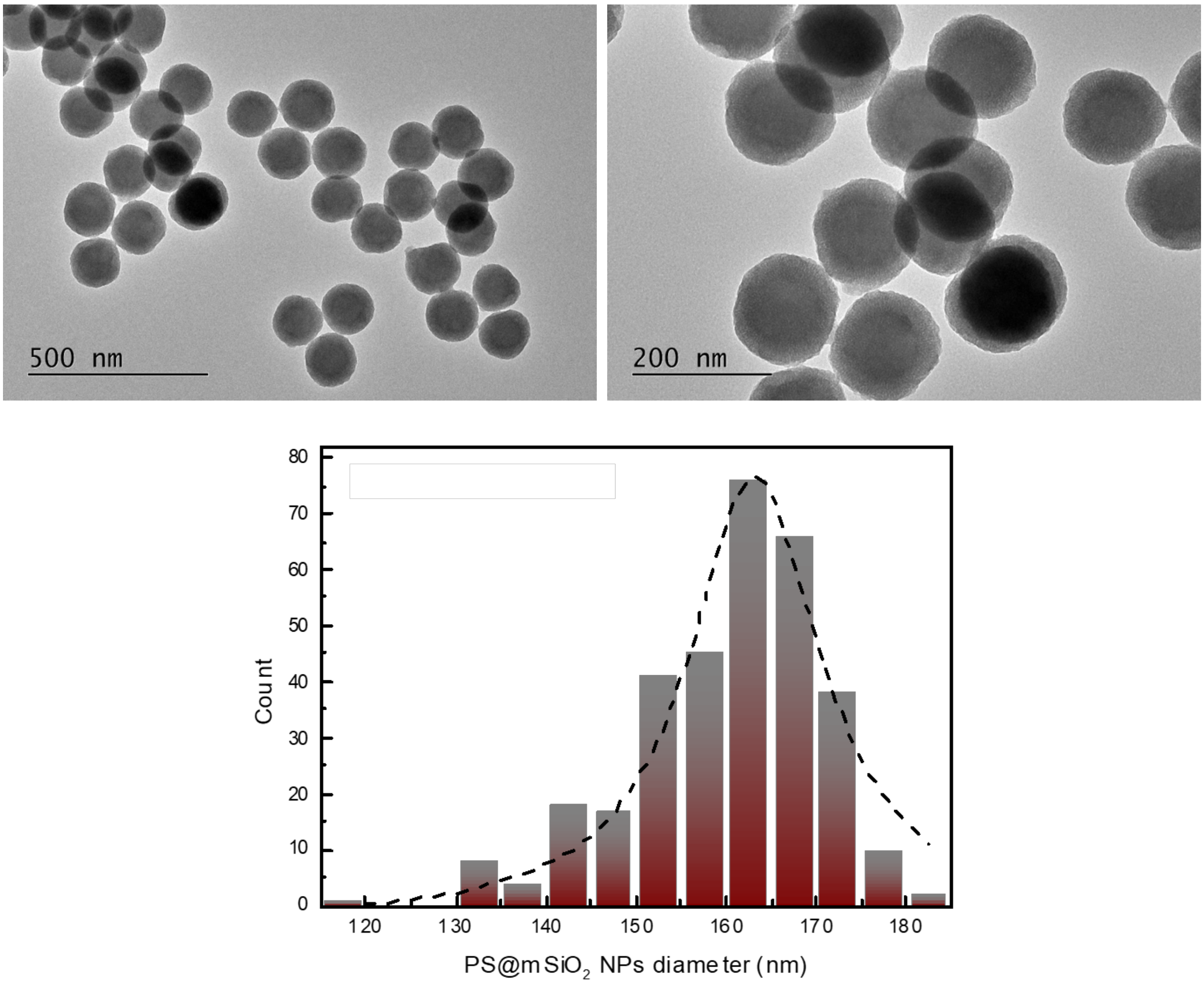

Figure S1. TEM images and sample distribution of sample **A**.

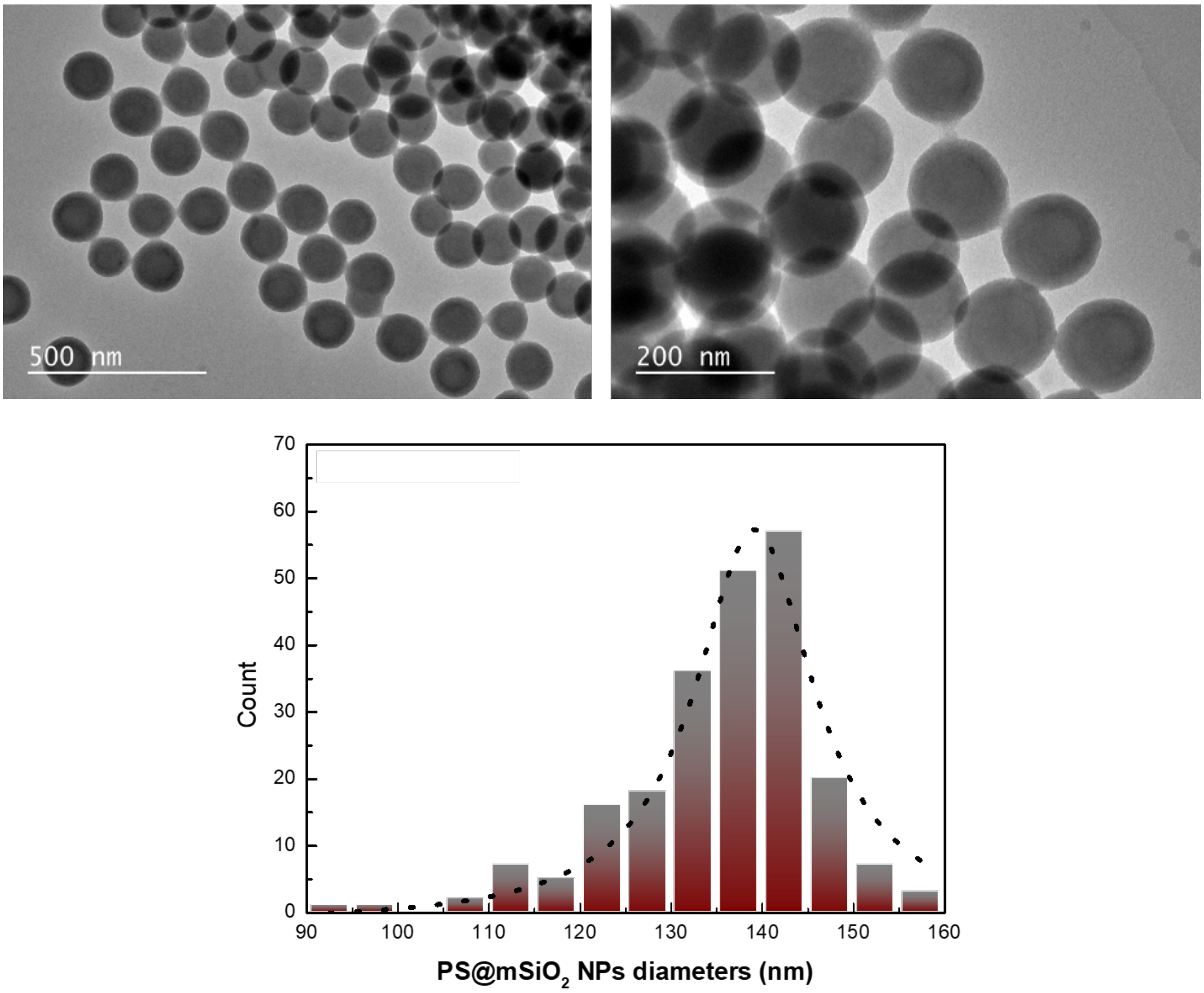

Figure S2. TEM images and size distribution of sample **B**.

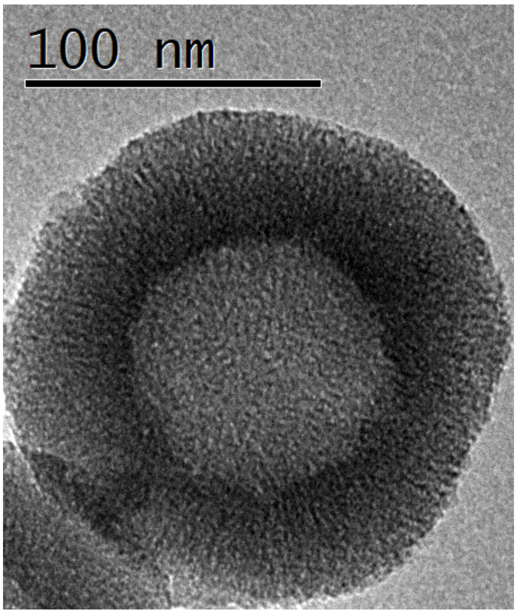

Figure S3. TEM image of a particle after calcination of sample A to remove the PS core and CTAB in the silica pores providing higher contrast to visualized the shell thickness.

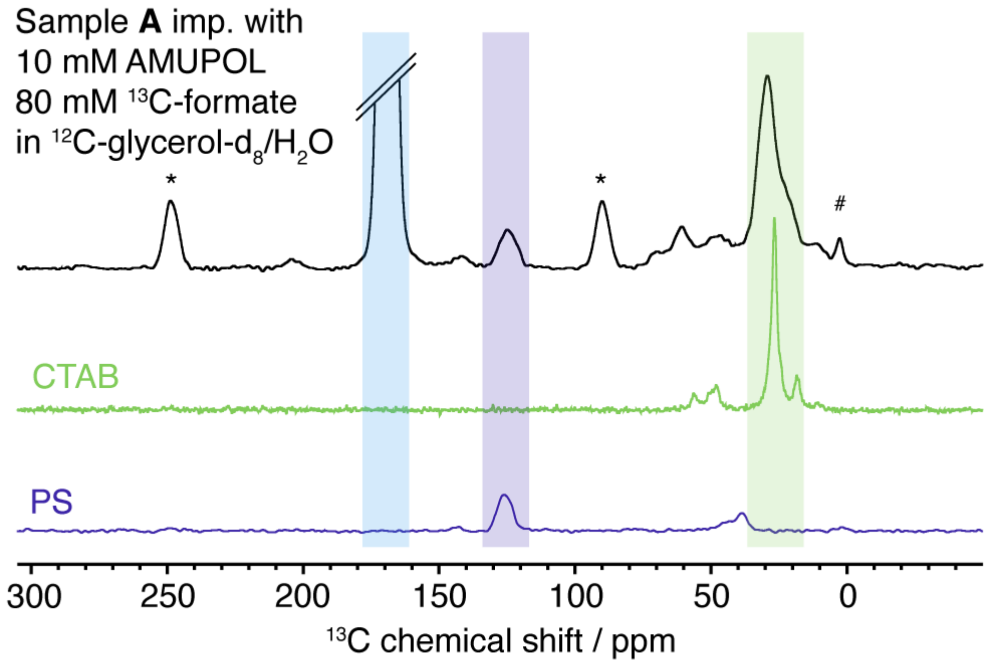

Figure S4. ^{1}H-^{13}C DNP CPMAS of (black) sample **A** impregnated with 10 mM AMUPOL and 80 mM ^{13}C-formate in ^{12}C-glycerol-d$_8$/H_2O ; (green) CTAB in silica matrix ; (blue) PS. All spectra are recorded at 9.4 T (400 MHz ^{1}H frequency), MAS rate of 8 kHz, temperature of ~100 K. Sample **A** is recorded under μwave irradiation, * denotes spinning sidebands and the peak at 170 ppm is ^{13}C signal of ^{13}C-formate, # is the ^{13}C signal of the silicon plug used to seal the rotor. The three rectangles indicate the three integration regions to obtain the (left to right) solvent/formate (light blue), PS (dark blue), and CTAB (green) signal intensities.

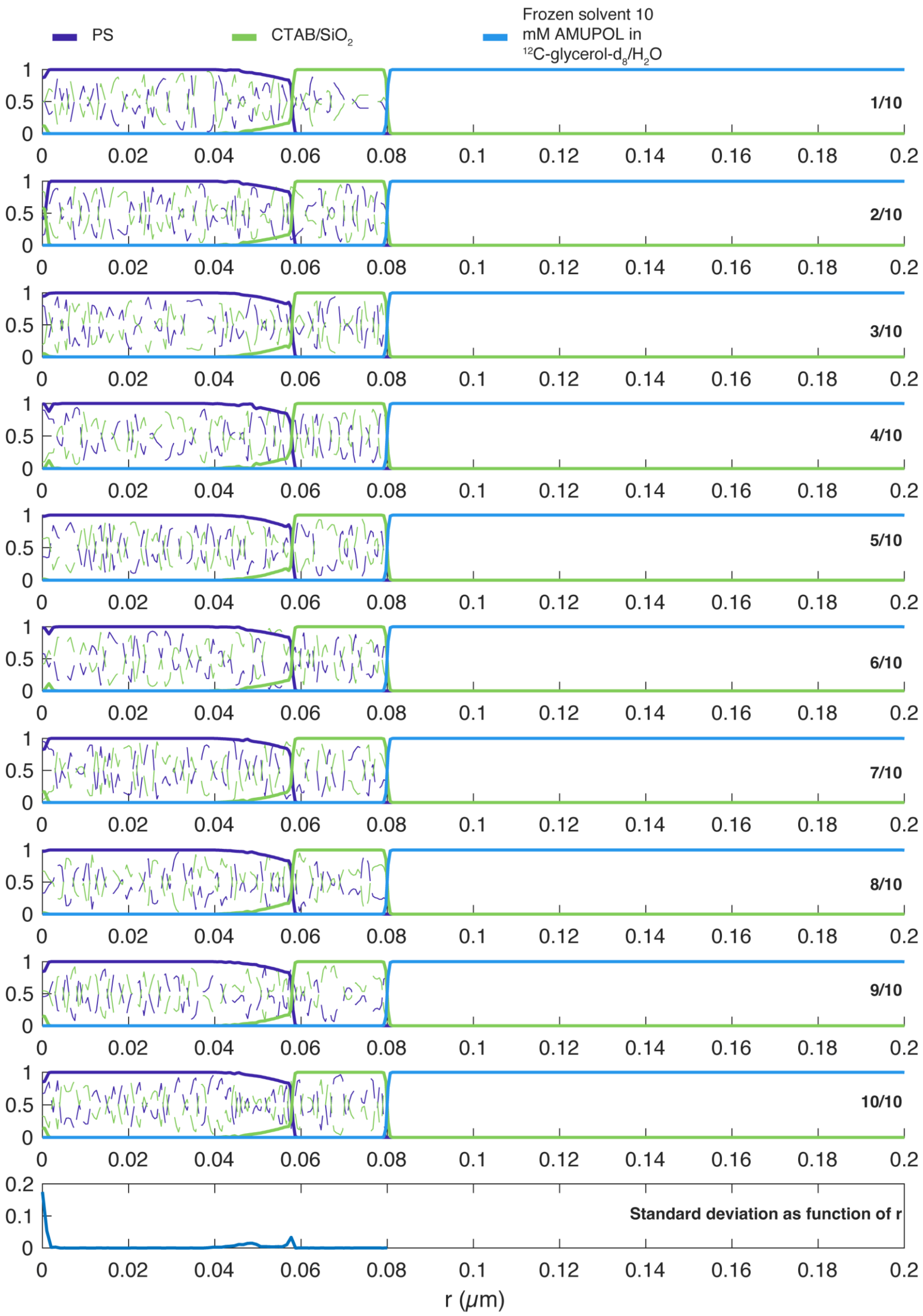

Figure S5. Error of the convergence procedure for sample **A**. 10% of noise is added on generated data from initial convergence and used to perform a new fit. It is repeated 10 times and reported from top to bottom. Dashed lines: initial guess composition; Plain line: composition after convergence. The last panel shows the standard deviation over 10 repetitions. The largest deviations are observed at the center and at the border between PS and SiO_2/CTAB regions. They represent less than 0.2 (at the maximum) variation in the composition.

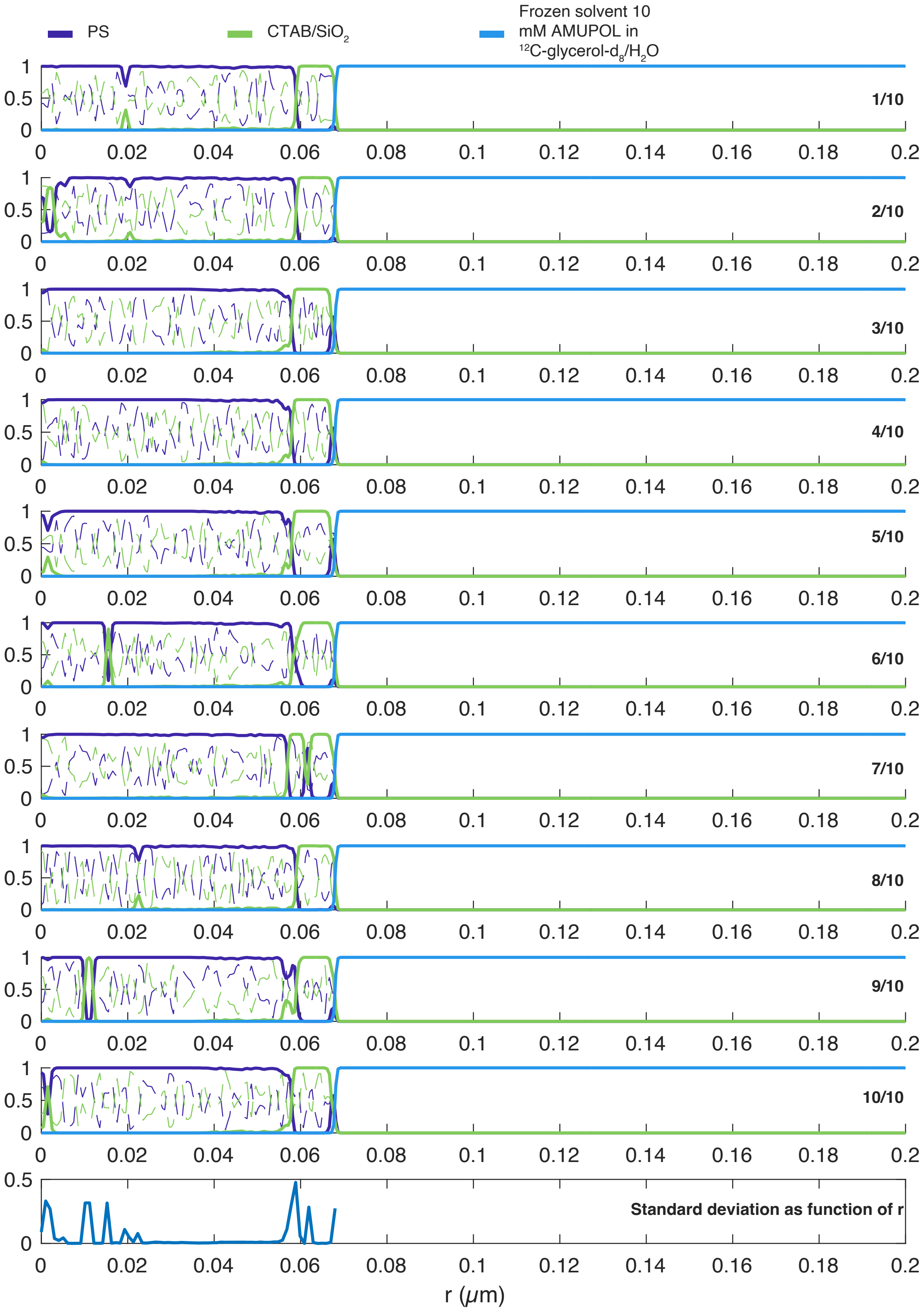

Figure S6. Error of the convergence procedure for sample **B**. 10% of noise is added on generated data from the initial convergence and used to perform a new fit. It is repeated 10 times and reported from top to bottom. Dashed lines: initial guess composition; Plain line: composition after convergence. The last panel shows the standard deviation over 10 repetitions.

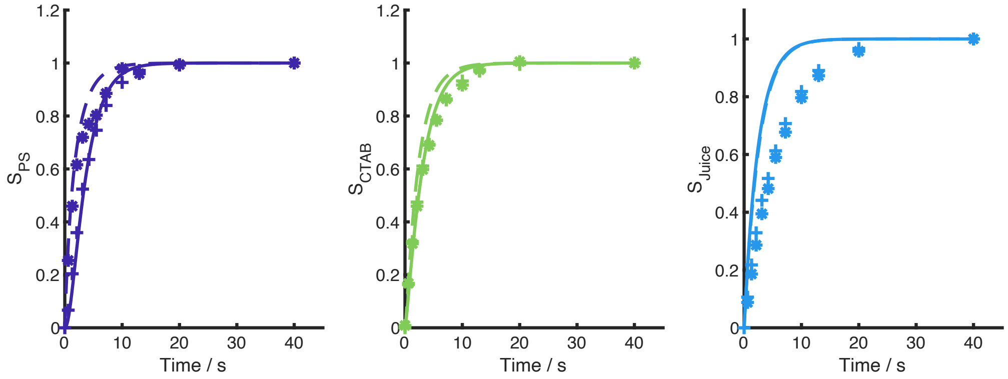

Figure S7. Signal build-up of PS, CTAB and Juice signals of sample **A**, normalized by taking the point at the longest time (ie. 40 s) equal to 1. Experimental build-up: asterisk points (*) are the signal without µwaves; cross points (+) are the signal with µwaves. Simulated build-up from the final composition after convergence: dashed lines are built-up without µwaves, plain lines are built-up with µwaves.

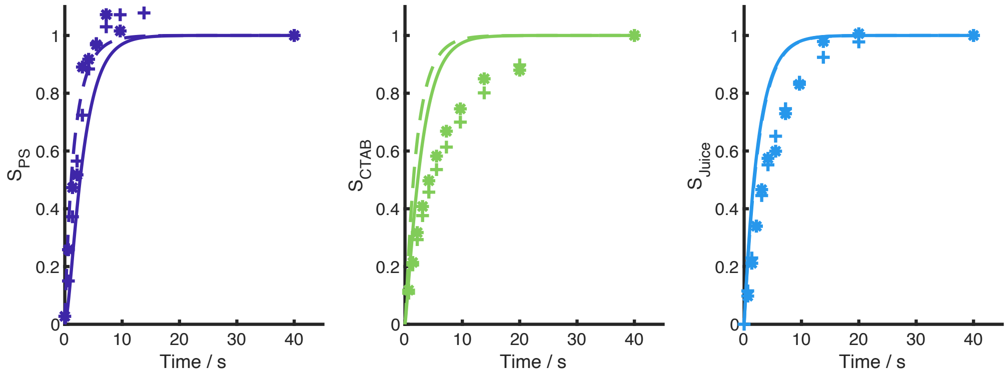

Figure S8. Signal build-up of PS, CTAB and Juice signals of sample **B**, normalized by taking the point at the longest time (ie. 40 s) equal to 1. Experimental build-up: asterisk points (*) are the signal without µwaves; cross points (+) are the signal with µwaves. Simulated build-up from the final composition after convergence: dashed lines are built-up without µwaves, plain lines are built-up with µwaves.

2. Experimental NMR parameters

**Sample A:** ^{1}H saturation-recovery detected via ^{1}H-^{13}C CPMAS	
^{1}H π/2 pulse	100 kHz
Number of saturation pulses	15
Delay between saturation pulses	3 ms
CP spin-lock	^{1}H: ramp from 68 kHz to 75 kHz<br>^{13}C: 70 kHz
CP contact time	1 ms
μwave source	klystron
Number of points in indirect dimension	12
Number of scans	μwave ON: 512<br>μwave OFF: 9616 (1st to 5th point of indirect dimension) and 1024 (6th to last point of indirect dimension)
Acquisition points	3988
Dwell time	5 μs
^{1}H decoupling	SPINAL-64 at 100 kHz
MAS rate	8 kHz

**Sample B:** ^{1}H saturation-recovery detected via ^{1}H-^{13}C CPMAS	
^{1}H π/2 pulse	100 kHz
Number of saturation pulses	20
Delay between saturation pulses	3 ms
CP spin-lock	^{1}H: ramp from 81 kHz to 90 kHz<br>^{13}C: 76 kHz
CP contact time	2 ms
μwave source	gyrotron
Number of points in indirect dimension	12
Number of scans	μwave ON: 128 (1st to penultima point of indirect dimension); 64 (last point)<br>μwave OFF: 1536 (1st to 6th point of indirect dimension); 512 (7th to penultima point of indirect dimension); 64 (last point)
Acquisition points	1994
Dwell time	10 μs
^{1}H decoupling	SPINAL-64 at 100 kHz
MAS rate	8 kHz

**Dry silica with pores filled with CTAB:** ^{1}H saturation-recovery detected via ^{1}H-^{13}C CPMAS	
^{1}H π/2 pulse	100 kHz
Number of saturation pulses	20
Delay between saturation pulses	3 ms
CP spin-lock	^{1}H: ramp from 43 kHz to 61 kHz<br>^{13}C: 54 kHz
CP contact time	2 ms
µwave source	n/a
Number of points in indirect dimension	16
Number of scans	16
Acquisition points	4096
Dwell time	10 µs
^{1}H decoupling	SPINAL-64 at 100 kHz
MAS rate	8 kHz
